# Triagem Direcionada da Hipercolesterolemia Familiar em 11 Pequenas Cidades Brasileiras: Uma Abordagem Eficaz para Detectar Agrupamentos de Indivíduos Afetados

**DOI:** 10.36660/abc.20220027

**Published:** 2022-04-07

**Authors:** Maria Cristina Oliveira Izar, Francisco A. H. Fonseca

**Affiliations:** 1 Universidade Federal de São Paulo São Paulo SP Brasil Universidade Federal de São Paulo, São Paulo, SP – Brasil

**Keywords:** Hiperlipoproteinemia Tipo II/genética, Testes Genéticos/métodos, Mutação com ganho de função, Diagnóstico Precoce, Doenças Genéticas Inatas

A hipercolesterolemia familiar (HF) é uma doença autossômica codominante associada a níveis elevados de LDL-colesterol e a doença cardiovascular aterosclerótica prematura.^1^ A condição é pouco reconhecida e devem ser implementadas ferramentas de rastreamento para melhorar o diagnóstico e promover o tratamento precoce.^2^ Os métodos de triagem incluem triagem universal, seletiva, em cascata, cascata reversa e triagem oportunística;^3,4^ no entanto, em cidades pequenas de certas regiões, onde o efeito fundador pode estar presente, a busca por novos casos a partir de indivíduos afetados pode ser uma opção interessante. O HipercolBrasil é um programa de rastreamento genético em cascata realizado no Instituto do Coração, com mais de 2.000 pacientes identificados com variantes patogênicas^5^ e, a partir desses resultados, foram selecionados casos índices (CI) de pequenas cidades com variantes patogênicas para amplificar a cascata. O programa realiza testes genéticos para HF em indivíduos com LDL-C ≥ 210 mg/dL confirmados em duas análises separadas: (CI)^6^ e em parentes de primeiro grau daqueles nos quais foram identificadas variantes patogênicas ou provavelmente patogênicas.

Em nosso país, as recomendações para testes genéticos seguem as Primeira Diretriz Brasileira de Hipercolesterolemia Familiar,^7^ endossadas pela Atualização da Diretriz Brasileira de Hipercolesterolemia Familiar – 2021.^8^

No artigo de Jannes et al.,^9^ os autores utilizaram um método de triagem direcionado aplicado a candidatos de 11 pequenas cidades brasileiras (com menos de 60.000 habitantes) com suspeita de alta prevalência de pessoas com HF. Selecionaram quatro cidades com suspeita de efeito fundador (Major Vieira, Papanduva, Lagoa do Mato e Passagem Franca); dois municípios em regiões com altas taxas de dislipidemia e infarto precoce, conforme descrito pelo banco de dados do Sistema Único de Saúde (Bom Despacho e Moema); e cinco cidades geograficamente próximas a outras cidades com alta prevalência de indivíduos com HF (Bambuí, Pimentas, Luz, Colinas e Buriti Bravo). Cento e cinco casos índice e 409 parentes de primeiro grau foram registrados nessas cidades. Usando tal abordagem, os autores encontraram 4,67 parentes por caso índice, o que foi significativamente maior (p < 0,0001) em comparação com a taxa geral do HipercolBrasil (1,59). Os métodos utilizados para confirmar o diagnóstico de HF foram *Next Generation Sequencing* (NGS) com um painel incluindo *LDLR, APOB, PCSK9, LDLRAP1, STAP1, LIPA, APOE, ABCG5 e ABCG8*. A triagem genética foi complementada por MLPA (*multiplex ligation-dependent probe amplification*) no gene *LDLR* para detectar variações no número de cópias do gene (CNVs) associadas à HF quando nenhuma mutação foi identificada. Este estudo mostrou que as taxas de detecção de HF foram maiores que as do programa HipercolBrasil e foram maiores nos municípios com efeitos fundadores. Por outro lado, em cidades próximas àquelas em que o efeito fundador esteve presente, a taxa de detecção de casos índice foi menor, consequentemente, o número de familiares afetados.

Em uma publicação anterior, os autores relataram que seu programa de rastreamento genético em cascata de HF poderia predizer a inclusão de novos familiares com base nas características do IC, informações úteis para a elaboração de abordagens de triagem melhores e mais eficazes para indivíduos sob risco.^10^No entanto, existe uma lacuna importante na percepção de risco, manejo do colesterol e outros aspectos relacionados à HF.^11^ A estratégia de triagem alvo em cidades pequenas pode ser eficaz, mas algumas questões devem ser levadas em consideração antes de escolher a cidade a ser triada. A participação do sistema de saúde local, a disponibilização de resultados laboratoriais prévios e a divulgação desta campanha em diferentes mídias sociais podem melhorar a adesão da população e melhores resultados.
