# Supplementary material for: Triagem Direcionada da Hipercolesterolemia Familiar em 11 Pequenas Cidades Brasileiras: Uma Abordagem Eficaz para Detectar Agrupamentos de Indivíduos Afetados
Source: Arq Bras Cardiol. 2022 Apr 7;118(4):678–9. [Article in Portuguese] doi: 10.36660/abc.20220027 (PMC9007013; doi:10.36660/abc.20220027)
Supplement: Translation

# Translation: Targeted Screening of Familial Hypercholesterolemia in 11 Small Brazilian Cities: An Effective Approach to Detect Clusters of Affected Individuals

**Authors:** Maria Cristina Oliveira Izar, Francisco A. H. Fonseca

**Affiliations:** 1 Brazil Universidade Federal de São Paulo, São Paulo, SP – Brazil

**Keywords:** Hyperlipoproteinemia Type II/genetics, Genetic Testing/methods, Gain of Function Mutation, Early Detection, Genetic Diseases, Inborn

Familial hypercholesterolemia (FH) is an autosomal codominant disease associated with high levels of LDL-cholesterol and premature atherosclerotic cardiovascular disease.^1^ The condition is underrecognized, and screening tools must be implemented to improve diagnosis and promote early treatment.^2^ The screening methods include universal, selective, cascade, reverse cascade, and oportunistic screening;^3,4^ however, in small cities in certain regions, where founder effects can be present, target search for affected individuals can be an interesting option. The HipercolBrasil is a genetic cascade screening program carried out in the Heart Institute, with more than 2,000 patients identified with pathogenic variants,^5^ and from these results, index cases (IC) from small cities with pathogenic variants were selected to amplify the cascade. The program performs genetic tests for FH in individuals with confirmed LDL-C ≥ 210 mg/dL in two separate analyses: (IC)^6^ and in first-degree relatives of those in whom pathogenic or likely pathogenic variants were identified.

In our country, the recommendations for genetic testing follow the First Brazilian Guideline for Familial Hypercholesterolemia,^7^ endorsed by the Update of the Brazilian Guideline for Familial Hypercholesterolemia – 2021.^8^

In the article by Jannes et al.,^9^ the authors used a targeted screening method applied to candidates from 11 small Brazilian cities (with less than 60,000 inhabitants) with a suspected high prevalence of people with FH. They selected four cities with suspected founder effect (Major Vieira, Papanduva, Lagoa do Mato, and Passagem Franca); two cities in regions with high rates of dyslipidemia and early myocardial infarction, as described by the National Health System database (Bom Despacho and Moema); and five cities that were geographically close to other cities with a high prevalence of individuals with FH (Bambuí, Pimentas, Luz, Colinas, and Buriti Bravo). One-hundred and five index cases and 409 first-degree relatives were enrolled from those cities. Using such approach, the authors found 4.67 relatives per index case, which was significantly higher (p < 0.0001) compared with the general HipercolBrasil rate (1.59). The methods used to confirm the diagnosis of FH were next-generation sequencing (NGS) with a panel including *LDLR, APOB, PCSK9, LDLRAP1, STAP1, LIPA, APOE, ABCG5* and *ABCG8 *genes. The genetic screening was complemented by MLPA (multiplex ligation-dependent probe amplification) in the *LDLR* gene to detect gene copy number variations (CNVs) associated with FH when no mutation was identified. This study has shown that the rates of FH detection were higher than the HipercolBrasil program and were also higher in cities with founder effects. On the other hand, in cities close to those in which founder effects were present, the detection rate of index cases was lower, consequently, the number of affected relatives.

In a previous publication, the authors reported that their FH cascade screening program could predict family enrollment based on IC features, useful information for devising better and more effective screening approaches for at-risk individuals.^10^ However, there is an important gap in risk perception, cholesterol management, and other aspects related to FH.^11^ The strategy of target screening in small cities can be effective, but some issues must be taken into account before choosing the city to screen. The participation of the local health system, availability of previous laboratory results, and advertisement of this campaign in different social media may improve adherence of the population and better results.
